# American Quarterly Journal of Agriculture and Science

**Published:** 1845-09

**Authors:** 


*American Quarterly Journal of Agriculture and Science.*— This is a quarterly journal published in the city of Albany, and conducted by Dr. E. Emmons and Dr. A. J. Prime. Each number contains about 200 octavo pages of original and selected matter, on scientific and practical subjects connected with Agriculture, Horticulture, and the kindred branches. The size of the work admits of amplified discussion of the various subjects treated of, and many articles highly interesting and instructive are to be found among the original papers. Geology, Entomology and Chemistry receive, in the work, the attention they deserve. Among other papers valuable to agriculturists, we may notice a series, continuing on in several numbers, on "Insects injurious to vegetation." From the minuteness of the descriptions, and the beautifully colored drawings accompanying the paper, no one can fail to recognize the little devastators. The mechanical execution of the work is excellent. We take great pleasure in recommending this publication to those of our readers who are interested in the subject of Agriculture, as both valuable and cheap. Terms $3,00 a year.—Ed.

